# Central Obesity and Associated Factors among Adult Patients on Antiretroviral Therapy (ART) in Armed Force Comprehensive and Specialized Hospital, Addis Ababa, Ethiopia

**DOI:** 10.1155/2021/1578653

**Published:** 2021-08-30

**Authors:** Adnan Kemal, Mohammed Ahmed, Melese Sinaga Teshome, Kalkidan Hassen Abate

**Affiliations:** ^1^Department of Public Health, College of Health Science, Defense University, Addis Ababa, Ethiopia; ^2^Department of Public Health, College of Health Science, Woldia University, Woldia, Ethiopia; ^3^Department of Nutrition and Dietetics, Jimma University, Jimma, Ethiopia

## Abstract

**Background:**

Similar to the general population, the prevalence of central obesity is increasing among HIV-infected persons. There are little data on the burden of abdominal obesity using the waist-to-hip ratio measurement in HIV-infected patients in resource-limited settings, including Ethiopia. Therefore, this study aimed to assess the prevalence and associated factors of central obesity among HIV patients taking ART in an armed force comprehensive and specialized hospital, Addis Ababa, Ethiopia.

**Methods:**

A cross-sectional study was conducted from March to April 2018. A systematic sampling method was used to select 353 study participants. Pretested World Health Organization stepwise questionnaire, document review, and anthropometric and biochemical measurements were used to collect data on different variables under the study. The collected data were entered into EpiData version 3 and analyzed by SPSS version 21. An adjusted odds ratio with 95% CI was considered to declare a statistically significant association.

**Results:**

The prevalence of central obesity in this study was 71.7% (95% CI: 67%–76.4%). Besides, the odds of central obesity were associated with being female (AOR: 85.6; 95% CI: 20.09, 364.6), among merchants (AOR: 18.8; 95% CI: 1.39, 255.7), CD4 count <200 cells/mm^3^ (AOR: 0.03; 95% CI: 0.007, 0.160), among respondents taking AZT + 3TC + EFV-based ART regimen (AOR: 8.73; 95% CI: 1.33, 57.17), ABC + 3TC + ATV/r-based regimen (AOR: 0.18; 95% CI: 0.03, 0.94), increased BMI (AOR: 3.50; 95% CI: 1.36, 3.89), and abnormal blood pressure (AOR: 2.53; 95%: 1.13, 5.67).

**Conclusion:**

It is possible to conclude that central obesity is a huge public health problem among the HIV-infected population in the study area. Being female, increased BMI, low CD4 count, AZT + 3TC + EFV, ABC + 3TC + ATV/r-based regimen, and abnormal blood pressure were associated with central obesity. Therefore, adequate attention must be paid to primary and secondary control of these factors to reduce the prevalence of abdominal obesity among HIV-infected patients.

## 1. Introduction

Effective viral suppression through the prolonged use of antiretroviral treatment (ART) has dramatically improved prognosis and survival evidenced by declining the toll of AIDS-related deaths [1]. In Ethiopia, an estimated 0.9% of men and women aged 15–49 years have been infected with the Human Immune Virus (HIV), and 71% of the eligible patients are currently on antiretroviral therapy (ART) [2].

People living with treated HIV infection today enjoy greater life expectancy and thus are more exposed to the obesogenic environment and accumulate age-related cardiometabolic risk factors [1, 3]. HIV-infected patients appear to frequently develop increased visceral fat and metabolic syndrome after starting ART regimen [4, 5].

Studies showed that obesity affects 2 in 5 HIV-infected women and 1 in 5 HIV-infected men compared with men and women in the general population, respectively [6]. HIV-infected patients had more abdominal visceral fat than extremity fat and demonstrated a higher waist-to-hip ratio (WHR) compared to the control population [7–9]. Similar to the general population, the prevalence of central obesity (the localized accumulation of adipose tissue in the abdomen irrespective of proportion to total body fat) is increasing among HIV-infected persons, for instance, 4% in the United States [10], 12.6% in Tanzania [11], 36.5% in Malaysia [12], and 45.7% in Brazil [13].

Different factors were implicated for central obesity such as female gender [14], advanced age (age of 40–49 years and ≥50 years) [10, 12, 14], higher CD4+ lymphocyte count (>350 cells/mm³) [10, 14], higher fasting plasma glucose [12], and higher body mass index (BMI) [12]. Evidence also showed that, for every increase of 10 g of lipid intake, the odds of central obesity increased 1.28 times. Carbohydrate consumption showed a negative association with central obesity [13].

Central obesity has been shown to correlate strongly with metabolic syndrome, hyperuricemia, and the progression of coronary calcium scores, which is a marker of cardiovascular disease (CVD) [15–18]. Also, abdominal adiposity is directly related to accelerating the aging process of the immune system [5], increased risk of dementia, fatty liver disease [5, 18], obstructive sleep apnea [19], reduced self-reported quality of life, decreased ART adherence, and a fear of disclosure of HIV status [20].

Currently, WHR is not so often used for evaluating central adiposity in the general population, and in its place waist circumference is the most widespread anthropometric measurement used. Besides, minimal agreement with BMI in identifying HIV/AIDS patients with increased cardiometabolic risk was reported [21]. Hence, WHR is useful in detecting possible signs of excess fat deposition in those infected with HIV [7, 13, 18].

Even though numerous studies have been conducted on central obesity concerning HIV infection and ART in developed countries, there are few data on the burden of abdominal obesity using WHR in HIV-infected patients in resource-limited settings, including Ethiopia in general, and no study was done in the study area in particular. Therefore, this study aimed to assess the prevalence and associated factors of central obesity among HIV-infected patients taking ART in an armed force comprehensive and specialized hospital, Addis Ababa, Ethiopia, for the first time.

## 2. Methods and Materials

### 2.1. Study Setting and Design

A cross-sectional study was conducted from March to April 2018 at an armed force comprehensive and specialized hospital, Addis Ababa, which is the capital city of Ethiopia, the seat of the African Union, and economic commission for Africa. The hospital has been delivering health services to the defense forces including civilians in the Ministry of Defense. The hospital launched a free ART program at the end of 2003. The total number of subjects ever enrolled for HIV care before the study period was 4,329 (4,053 adults and 276 children). From the total numbers of adult subjects ever enrolled for HIV care, 2,263 adults were currently on ART in the hospital; from this, 1750 subjects were followed up during the study period.

Individuals living with HIV/AIDS, aged ≥18 years, who had been receiving ART for at least 6 months or more and was able to communicate and provide informed consent were included in the study. Pregnant women and individuals who had a physical deformity of the waist and hip were excluded from the study.

### 2.2. Sample Size Determination and Sampling Procedure

The sample size was calculated by using a single population proportion formula by taking a 50% proportion of central obesity among HIV patients on ART, since there was no study conducted in Ethiopia using WHR, 95% CI, and 5% margin of error, and the minimum final sample size becomes 384. The systematic sampling method was employed to select the study participants every 5th interval after preparing a sampling frame based on patient order when coming to the hospital for follow-up.

### 2.3. Definition of Central Obesity

We used the European cutoff point to interpret the waist-to-hip circumference ratio measurement for sub-Saharan African people according to International Diabetes Federation (IDF) recommendations; central obesity was defined as waist-to-hip ratio (WHR) ≥0.94 cm for men and ≥0.80 cm for women [22, 23].

### 2.4. Data Collection Instrument and Procedure

#### 2.4.1. Face-to-Face Interview

Data were collected using a structured questionnaire adapted from the WHO stepwise approach to chronic disease risk factor surveillance [24]. The questionnaire contained sociodemographic characteristics (age, sex, marital status, educational status, and occupation). Questions related to depression (by using the Beck Depression Inventory score) and individual dietary diversity score (DDS) were added to supplement the WHO STEPwise approach. WHO stage during ART initiation, duration on ART, baseline CD4 count, viral load count, and type of ART regimen were collected from the patient's health record.

#### 2.4.2. Anthropometric Measurements

Weight, height, waist circumference, and hip circumference were measured using calibrated equipment. Weight was measured using the SECA 704 medical weighing scale. Body mass index was calculated by dividing a person's weight in kilograms by the square of their height in meters. Waist circumference was measured at the horizontal plane which corresponds to the midpoint between the anterior superior iliac spine and the lower costal margin at the midclavicular line. Also, hip circumference measurement was done for participants by using a SECA 200 cm measuring tape. To improve the reliability of measurement for all anthropometric measurements, two readings were taken and the average of the two readings was recorded as the final measurement of the respondent.

#### 2.4.3. Blood Pressure Measurements

Blood pressure (BP) was measured using a standard adult arm cuff of mercury type sphygmomanometer after 5 min rest in the clinic by the nurses working in the ART clinic. To improve the reliability of measurement, three readings were taken with 5 min intervals and the average of the three readings was recorded as the final BP of the patient. But if the difference between the two readings was greater than 5 mmHg, a third measurement was taken and recorded as the final BP of the patient.

#### 2.4.4. Biochemical Measurements

Fasting blood was collected and analyzed by qualified laboratory technologists for biochemical measurements. The study subjects were requested to return to the clinics to provide fasting blood samples (4 ml) for blood glucose, lipid profile (triglyceride, total cholesterol levels, HDL-c, and LDL-c), and current CD4 count measurements the next day in case if they had eaten and the test was done in next morning. All measurements were analyzed in the Cobas Mira Plus spectrophotometer (Roche Diagnostics) equipped with calibration filters and DIASYS serum control.

### 2.5. Data Collection Procedure

The questionnaire was first written in English and then translated into Amharic and back to English by linguistic experts, and it was tested before use. A research team of nine individuals (4 ART nurses, 1 public health expert, 2 laboratory technicians, and 2 lab technologists) collected data on sociodemographic characteristics and performed clinical, behavioral, anthropometric, blood pressure, and biochemical measurements. A supervisory team (1 public health expert and 1 lab technologist) including the principal investigator was in place during the data collection. Both the interviewers and supervisors were trained for two days on the objective of the study, methods on how to collect data and interviewing approach, anthropometric measurement, biochemical measurement, and blood pressure measurement.

### 2.6. Data Quality Assurance

The proper functioning of instruments, laboratory reagents, and technical performance was checked by using quality control samples (serum pool). Standard operating procedures (SOPs) were followed starting from sample collection up to result reporting. All laboratory procedures were handled by laboratory technologists. Before data analysis, cleaning was done.

### 2.7. Data Processing and Analysis

The data were collected, cleaned, and checked for completeness and consistency manually. They were coded and entered into EpiData version 3.1. Then they were exported to SPSS (version 21.0) for further cleaning and statistical analysis. Descriptive statistics such as frequency, percentage, mean, and standard deviation were used to describe characteristics of the study population. Bivariate analysis was done using binary logistic regression, a variable of interest, and all covariate variables which had an association with the outcome variables at a *p* value of less than 0.25 were selected for multivariable binary logistic regression analysis. Multivariable binary logistic regression models were used to isolate independent predictors of central obesity. A two-sided *p* value <0.05 along with an adjusted odds ratio (AOR) of 95% confidence interval was considered to declare statistically significant associations. The fitness of the model was checked using the Hosmer–Lemeshow test and the model was fitted having a *p* value of 0.57.

### 2.8. Ethical Consideration

Ethical clearance was obtained from the Institutional Review Board of the Institute of Health, Jimma University, and then permission was obtained from the armed force comprehensive and specialized hospital review board and ART clinic head. Written informed consent was secured from each study subject after the benefit, risk, and danger of participation were explained. Confidentiality was assured and maintained. A thumbprint or signature was used on the consent form. If the participants were diagnosed with lipid abnormality, fasting glucose impairment, and increased blood pressure, he/she was linked to the hospital for chronic care proper treatment, and follow-up of this disease as well coincides with HIV/ART disease. Every laboratory investigation was done according to the requirement of the patient. Health education on risk factors, consequences, and complications were provided to all of the participants after the compilation of data collection.

## 3. Results

### 3.1. Descriptive Statistics of the Study Participants

A total of 353 respondents were included and analyzed in this study, yielding a response rate of 97%. 172 (48.7%) respondents were female. The mean age of the participants was 44.6 ± 8.85 years. Regarding education, 205 (58.1%) respondents have attended primary/secondary education. Concerning occupation, 87 (24.6%) of them were employed. Looking upon marital status, 248 (70.3%) respondents were married (Table 1).

### 3.2. Clinical and Behavioral Characteristics of Study Participants

117 (33.1%) respondents were found to be at WHO clinical stage II at baseline. After ART initiation, 209 (59.2%) of them had >107 months of ART duration at the time of the survey. Moreover, 185 (52.4%) respondents were found in TDF + 3TC + EFV-based ART regimen. The proportions of the participants who smoked cigarettes, drank alcohol, and chewed khat were 13 (3.7%), 131 (37.1%), and 12 (3.4%), respectively. 288 (81.6%) respondents were depressed at the time of the survey (Table 2).

### 3.3. Laboratory and Anthropometry-Related Parameters among the Study Participants

The mean levels of TC, LDL, and HDL were 1.99.3 (±46.3), 117.6 (±56.4), and 49.1 (±14.82) mg/dl, respectively. The mean baseline and current CD4 counts were 182.58 (±144.19) and 455.69 (±262.84), respectively. The mean recent viral load was 10895.9 ± 79634. Likewise, the mean of the FBS level of the study participants was 108.55 (±48.879) mg/dl. As well, the mean waist circumference and hip circumference of the participants were 88.8 (±9.97) and 94.68 (±9.28) cm respectively (Table 3).

### 3.4. Prevalence of Central Obesity among HIV Patients Who Received ART at Armed Force Comprehensive and Specialized Hospital

The prevalence of central obesity in this study was 71.7% (95% CI: 67%–76.4%). 155 (43.9%) female respondents developed central obesity. Besides, 138 (39.1%) respondents who developed central obesity attended primary/secondary education. As well, 94 (26.6%) respondents who developed central obesity were found to be at WHO clinical stage II at baseline. Furthermore, 154 (43.6%) respondents who developed central obesity had >107 months of ART duration. 125 (35.4%) respondents who developed central obesity were found in TDF + 3TC + EFV-based ART regimen. Among the participants who developed central obesity, 92 (26.1%) consumed alcohol. 129 (36.5%) of the respondents had abnormal total cholesterol (TC), and 172 (48.7%) had abnormal low-density lipoprotein (LDL) (Table 4).

### 3.5. Factors Associated with Central Obesity among HIV Patients Who Received ART at Armed Force Comprehensive and Specialized Hospital, Ethiopia

All the variables were entered into multivariate binary logistic regression analysis by the enter method of model selection. After adjusting for potential confounders by logistic regression, sex, occupational status, type of ART regimen, current CD4 counts, BMI, and abnormal blood pressure were associated with central obesity.

In this study, the odds of central obesity were 85.6 (AOR: 85.6; 95% CI: 20.09, 364.6) times higher among females compared to males. Likewise, the odds of central obesity were 18.8 (AOR: 18.8; 95% CI: 1.39, 255.7) times higher among merchants compared to housewives.

Moreover, the odds of central obesity were decreased by 97% (AOR: 0.03; 95% CI: 0.007, 0.160) among participants who had a CD4 count <200 cells/mm^3^ compared to respondents who had a CD4 count >500 cells/mm^3^.

Likewise, the odds of central obesity were 8.73 (AOR: 8.73; 95% CI: 1.33, 57.17) times higher among respondents taking AZT + 3TC + EFV compared to AZT + 3TC + NPV-based ART regimen. However, participants taking ABC + 3TC + ATV/r-based regimen were 82% (AOR: 0.18; 95% CI: 0.03, 0.94) protective against central obesity compared to AZT + 3TC + NPV-based ART regimen.

As the BMI of the participants increased by 1 kg/m^2^, the odds of central obesity increased by 3.5 (AOR: 3.50; 95% CI: 1.36, 3.89).

The odds of central obesity were 2.53 (AOR: 2.53; 95%: 1.13, 5.67) times higher among participants who had abnormal blood pressure when compared to their counterparts (Tables 5 and 6).

## 4. Discussion

In this study, the prevalence of central obesity was 71.7% (95% CI: 67%–76.4%). This finding is higher than those in studies conducted elsewhere, for instance, 4% in the United States [10], 12.6% in Tanzania [11], 36.5% in Malaysia [12], and 45.7% in Brazil [13]. This discrepancy may be due to the type used to measure central obesity and sample size variation, in which the current study utilized a large sample size and WHR, which early detected central obesity among HIV-infected population.

The present study also revealed that sex, occupational status, type of ART regimen, current CD4 counts, BMI, and abnormal blood pressure were associated with central obesity.

The odds of central obesity were higher among females compared to males. This finding is supported by studies conducted in Brazil [14], Nigeria [25], and Kenya [26]. This could be expounded by HIV-infected women who had a higher waist-to-hip ratio (WHR) than the control population [7]. Also, men were more likely to progress to overweight and women to obesity [9].

Besides, women have substantially more total adipose tissue than men, and these whole‐body sex differences are complemented by major differences in tissue distribution [27].

The odds of central obesity were higher among merchants than among housewives. This may be related to having a high-income result in sedentary life which was associated with central obesity [25].

The study further showed that increased BMI results in increased odds of central obesity. This finding is evidenced by studies done in Malaysia [12]. This could be explained by changes in the waist and hip circumferences correlated directly with weight changes [27].

In this study, a higher CD4 count increased the odds of central obesity. This finding is similar to those in other studies conducted elsewhere [14, 25]. However, a systematic review showed that central obesity is evident among patients with CD4 counts <350 cells/mm^3^ but not among patients with higher CD4 counts [17]. The result found reaffirms the hypothesis that being overweight is linked to a better immune status, but since the studies are controversial, investigation in future research is warranted.

Abnormal blood pressure results in higher odds of central obesity. This finding is in line with studies done in Cameroon [28] and Indonesia [29]. This could be explained by having a close association between hypertension and dyslipidemia. Studies showed that hypertensive patients compared with normotensives have higher serum levels of TC, TG, and LDL, while HDL levels were lower [29].

Furthermore, the odds of central obesity were higher among respondents taking AZT + 3TC + EFV compared to AZT + 3TC + NPV-based ART regimen. [30]. Likewise, the odds of central obesity were higher among respondents taking AZT + 3TC + EFV compared to AZT + 3TC + NPV-based ART regimen. However, participants taking ABC + 3TC + ATV/r-based regimen were 82% protective against central obesity compared to AZT + 3TC + NPV-based ART regimen. Similar findings were found in Silchar [31]. Efavirenz (EFV) and zidovudine (AZT) have already been reported as causative of metabolic syndrome by increased TG and LDL-c levels but decreased HDL-c levels [32].

### 4.1. Strength and Limitation of the Study

The strength of the present study is that central obesity is assessed using WHR, which is useful in early detecting possible signs of excess fat deposition in those infected with HIV. Although findings in this study are useful for policy, there are some noteworthy limitations. For example, the data were collected in a cross-sectional study; therefore, our study may not assign causation.

## 5. Conclusions

From the findings of the study, it is possible to conclude that central obesity is a huge public health problem among the HIV-infected population in the study area. Being female, increased BMI, low CD4 count, AZT + 3TC + EFV, ABC + 3TC + ATV/r-based regimen, and abnormal blood pressure were associated with central obesity. Therefore, adequate attention must be paid to primary and secondary control of these factors to reduce the prevalence of abdominal obesity among HIV-infected patients.

## Figures and Tables

**Table 1:** Sociodemographic profile of the HIV patients who received ART at armed force comprehensive and specialized hospital, Ethiopia, 2018 (*n* = 353).

Variables	Category	Frequency (%)
Sex	Female	172 (48.7)
	Male	181 (51.3)

Age (mean ± SD)	Mean: 44.6 ± 8.85 years	

Educational status	Illiterate	50 (14.2)
	Primary/secondary	205 (58.1)
	College and above	98 (27.8)

Occupation	Housewife	50 (14.2)
	Employer	87 (24.6)
	Merchant	52 (14.7)
	Pensioners	19 (5.4)
	Unemployed	145 (41.1)

Marital status	Married	248 (70.3)
	Never married	31 (8.8)
	Widowed	34 (9.6)
	Divorced	40 (11.3)

**Table 2:** Clinical and behavioral characteristics of HIV patients who received ART at armed force comprehensive and specialized hospital, Ethiopia, 2018 (*n* = 353).

Variables	Category	Frequency (%)
WHO clinical stage	Stage I	57 (16.1)
	Stage II	117 (33.1)
	Stage III	109 (30.9)
	Stage IV	70 (19.8)

Duration on ART	<59 months	48 (13.6)
	60–107 months	96 (27.2)
	>107 months	209 (59.2)

Type of regimen	AZT + 3TC + NPV	68 (19.3)
	AZT + 3TC + EFV	30 (8.5)
	TDF + 3TC + EFV	185 (52.4)
	TDF + 3TC + NPV	37 (10.5)
	ABC + 3TC + ATZ	12 (3.4)
	ABC + 3TC + ATV/r	21 (5.9)

Smoked cigarette	Yes	13 (3.7)
	No	340 (96.3)

Consumed alcohol	Yes	131 (37.1)
	No	222 (62.9)

Physical activity	Yes	244 (69.1)
	No	109 (30.9)

Chewed khat	Yes	12 (3.4)
	No	341 (96.6)

Dietary diversity score (DDS)	Diversified	195 (55.2)
	Undiversified	158 (44.8)

Depression status	Yes	288 (81.6)
	No	65 (18.4)

**Table 3:** Laboratory and anthropometry-related parameters among HIV patients who received ART at armed force comprehensive and specialized hospital, Ethiopia, 2018 (*n* = 353).

Parameters	Mean ± SD
Total cholesterol (TC)	199.3 (±46.3) mg/dl
Low-density lipoprotein (LDL)	117.6 (±56.4) mg/dl
High-density lipoprotein(HDL)	49.1 (±14.82) mg/dl
Total triglycerides (TG)	137.6 (±78.9) mg/dl
Fasting blood glucose (FBS)	108.5 (±48.9) mg/dl
Waist circumference	88.8 (±9.97) cm
Hip circumference	94.68 (±9.28) cm
Body mass index	23.17 (±4.094) kg/m^2^
Baseline CD4 counts	182.58 (±144.19) cells per mm^3^
Current CD4 counts	455.69 (±262.84) cells per mm^3^
Recent viral load	10895.9 ± 79634 copies per ml

**Table 4:** Prevalence of central obesity among HIV patients who received ART at armed force comprehensive and specialized hospital, Ethiopia, 2018 (*n* = 353).

Variables	Category	WHR	
		Abnormal (%)	Normal (%)
Sex	Female	155 (43.9)	17 (4.8)
	Male	98 (27.8)	83 (23.5)

Educational status	Illiterate	42 (11.9)	8 (2.3)
	Primary/secondary	138 (39.1)	67 (19.1)
	College and above	73 (20.7)	25 (7.1)

Occupational status	Housewife	45 (12.7)	5 (1.4)
	Employed	111 (31.4)	39 (11.0)
	Merchant	17 (4.8)	2 (0.6)
	Pensioners	60 (17.0)	50 (14.2)
	Unemployed	20 (5.7)	4 (1.1)

Marital status	Married	177 (50.1)	71 (20.1)
	Never married	20 (5.7)	11 (3.1)
	Widowed	30 (8.5)	4 (1.1)
	Divorced	26 (7.4)	14 (4.0)

WHO clinical stage	Stage I	34 (9.6)	23 (6.5)
	Stage II	94 (26.6)	23 (6.5)
	Stage III	79 (22.4)	30 (8.5)
	Stage IV	46 (13.0)	24 (6.8)

Duration on ART (months)	<59 months	34 (9.6)	14 (4.0)
	60–107 months	65 (18.4)	31 (8.8)
	>107 months	154 (43.6)	55 (15.6)

Type of regimen	AZT + 3TC + NPV	54 (15.3)	14 (4.0)
	AZT + 3TC + EFV	28 (7.9)	2 (0.6)
	TDF + 3TC + EFV	125 (35.4)	60 (17.0)
	TDF + 3TC + NPV	29 (8.2)	8 (2.3)
	ABC + 3TC + ATZ	5 (1.4)	7 (2.0)
	ABC + 3TC + ATV/r	12 (3.4)	9 (2.5)

Baseline CD4 counts (cells/mm^3^)	<200	173 (49.0)	60 (17.0)
	200–349	47 (13.3)	29 (8.2)
	350–499	20 (5.7)	10 (2.8)
	>500	13 (3.7)	1 (0.3)

Current CD4 counts (cells/mm^3^)	<200	17 (4.8)	16 (4.5)
	200–349	38 (10.8)	23 (6.5)
	350–499	61 (17.3)	17 (4.80)
	>500	137 (38.8)	44 (12.5)

Current viral load	≤1000 counts	234 (66.3)	86 (24.4)
	≥1001 counts	19 (5.4)	14 (4.0)

Smoked cigarette	Yes	9 (2.5)	4 (1.1)
	No	244 (69.1)	96 (27.2)

Consumed alcohol	Yes	92 (26.1)	39 (11.0)
	No	161 (45.6)	61 (17.3)

Physical activity	Yes	176 (49.9)	68 (19.3)
	No	77 (21.8)	32 (9.1)

Dietary/diversity score	Diversified	139 (39.4)	56 (15.9)
	Undiversified	114 (32.3)	44 (12.5)

Depression status	Normal	206 (58.4)	82 (23.2)
	Abnormal	47 (13.3)	18 (5.1)

Total cholesterol (TC)	Normal	129 (36.5)	62 (17.6)
	Abnormal	124 (35.1)	38 (10.80)

Low-density lipoprotein (LDL)	Normal	172 (48.7)	71 (20.1)
	Abnormal	81 (22.9)	29 (8.2)

High-density lipoprotein (HDL)	Normal	155 (43.9)	72 (20.4)
	Abnormal	98 (27.8)	28 (7.9)

Total triglyceride (TG)	Normal	175 (49.6)	76 (21.5)
	Abnormal	78 (22.1)	24 (6.8)

Blood pressure (BP)	Normal	149 (42.2)	69 (19.5)
	Abnormal	104 (29.5)	31 (8.8)

**Table 5:** A bivariate analysis using binary logistic regression to identify factors associated with central obesity among HIV patients who received ART at armed force comprehensive and specialized hospital, Ethiopia, 2018 (*n* = 353).

Variables	Category	COR (95% CI)	*p* value
Sex	Female	7.72 (4.32, 13.8)	0.002
	Male	1.00	

Age (years)		0.99 (0.96, 1.02)	0.23
Educational status	Illiterate	1.79 (0.74, 4.34)	0.52
	Primary/secondary	0.71 (0.41, 1.21)	0.06
	College and above	1.00	

Occupational status	Housewife	1.00	
	Employer	0.32 (0.12, 0.85)	0.02
	Merchant	0.94 (0.17, 5.34)	0.19
	Pensioners	0.13 (0.05, 0.36)	0.04
	Unemployed	0.56 (0.13, 2.29)	0.26

Marital status	Married	1.00	
	Never married	0.73 (0.33, 1.60)	0.15
	Widowed	3.00 (1.02, 8.85)	0.03
	Divorced	0.74 (0.37, 1.51)	0.06

WHO clinical stage	Stage I	1.00	
	Stage II	2.76 (1.37, 5.56)	0.01
	Stage III	1.80 (0.91, 3.50)	0.23
	Stage IV	1.30 (0.63, 267)	0.17

Duration on ART (months)	<59 months	1.00	
	60–107 months	0.86 (0.41, 1.84)	0.19
	>107 months	1.15 (0.58, 2.31)	0.08

Type of regimen	AZT + 3TC + NPV	1.00	
	AZT + 3TC + EFV	3.63 (0.77, 17, 11)	0.06
	TDF + 3TC + EFV	0.54 (0.28, 1.05)	0.13
	TDF + 3TC + NPV	0.94 (0.35, 2.50)	0.21
	ABC + 3TC + ATZ	0.18 (0.05, 0.67)	0.04
	ABC + 3TC + ATV/r	0.35 (0.12, 0.98)	0.02

Baseline CD4 counts (cells/mm^3^)	<200	0.22 (0.03, 1.73)	0.17
	200–349	0.12 (0.01, 1.00)	0.15
	350–499	0.15 (0.02, 1.35)	0.21
	>500	1.00	

Current CD4 counts (cells/mm^3^)	<200	0.34 (0.16, 0.73)	<0.001
	200–349	0.53 (0.29, 0.99)	0.24
	350–499	1.15 (0.61, 2.18)	0.11
	>500	1.00	

Current viral load (copies/ml)	≤1000 counts ≥1001 counts	1.00 0.50 (0.24, 1.04)	0.16

Body mass index (BMI)		1.23 (1.15, 1.32)	<0.001
Smoked cigarette	Yes	1.46 (0.48, 4.43)	0.18
	No	1.00	

Consumed alcohol	Yes	0.86 (0.55, 1.34)	0.30
	No	1.00	

Physical activity	Yes	1.00	0.17
	No	0.81 (0.50, 1.29)	

Dietary/diversity score	Diversified	1.05 (0.68, 1.61)	0.14
	Undiversified	1.00	

Depression status	Normal	1.00	
	Abnormal	0.76 (0.43, 1.35)	0.34

Total cholesterol (TC)	Normal	1.00	
	Abnormal	0.54 (0.35, 0.84)	0.02

Low-density lipoprotein (LDL)	Normal	1.00	
	Abnormal	1.83 (1.15, 2.89)	0.01

High-density lipoprotein (HDL)	Normal	1.00	
	Abnormal	1.62 (0.98, 2.69)	0.23

Total triglyceride (TG)	Normal	1.00	
	Abnormal	1.41 (0.83, 2.40)	0.12

Blood pressure (BP)	Normal	1.00	
	Abnormal	1.55 (0.95, 2.54)	0.21

Fasting blood glucose (FBS)		1.002 (0.99, 1.01)	0.16

**Table 6:** Multivariable binary logistic regression analysis to identify factors associated with central obesity among HIV patients who received ART at armed force comprehensive and specialized hospital, Ethiopia, 2018 (*n* = 353).

Variables	Category	COR (95% CI)	AOR (95% CI)	*p* value
Sex	Female	7.72 (4.32, 13.8)	85.6 (20.1, 364.6)	<0.001^*∗*^
	Male	1.00	1.00	

Age (years)		0.99 (0.96, 1.02)	1.01 (0.96, 1.06)	0.610
Educational status	Illiterate	1.79 (0.74, 4.34)	1.31 (0.37, 4.57)	0.673
	Primary/secondary	0.71 (0.41, 1.21)	0.48 (0.21, 1.07)	0.074
	College and above	1.00	1.00	

Occupational status	Housewife	1.00	1.00	
	Employer	0.32 (0.12, 0.85)	1.84 (0.36, 9.24)	0.46
	Merchant	0.94 (0.17, 5.34)	18.8 (1.39, 255)	0.03^*∗*^
	Pensioners	0.13 (0.05, 0.36)	1.31 (0.24, 7.10)	0.75
	Unemployed	0.56 (0.13, 2.29)	2.13 (0.19, 25.5)	0.55

Marital status	Married	1.00	1.00	
	Never married	0.73 (0.33, 1.60)	1.67 (0.5, 5.58)	0.405
	Widowed	3.00 (1.02, 8.85)	3.32 (0.59, 18.8)	0.175
	Divorced	0.74 (0.37, 1.51)	0.31 (0.09, 1.04)	0.058

WHO clinical stage	Stage I	1.00	1.00	
	Stage II	2.76 (1.37, 5.56)	2.59 (0.80, 8.33)	0.110
	Stage III	1.80 (0.91, 3.50)	2.20 (0.75, 6.42)	0.149
	Stage IV	1.30 (0.63, 267)	1.09 (0.34, 3.51)	0.879

Duration on ART (months)	<59 months	1.00	1.00	
	60–107 months	0.86 (0.41, 1.84)	0.49 (0.14, 1.73)	0.269
	>107 months	1.15 (0.58, 2.31)	0.35 (0.11, 1.15)	0.084

Type of regimen	AZT + 3TC + NPV	1.00	1.00	
	AZT + 3TC + EFV	3.63 (0.77, 17, 11)	8.73(1.33, 57.17)	0.024^*∗*^
	TDF + 3TC + EFV	0.54 (0.28, 1.05)	0.45(0.16, 1.29)	0.137
	TDF + 3TC + NPV	0.94 (0.35, 2.50)	0.51(0.10, 2.47)	0.401
	ABC + 3TC + ATZ	0.18 (0.05, 0.67)	0.27(0.02, 2.90)	0.278
	ABC + 3TC + ATV/r	0.35 (0.12, 0.98)	0.18(0.03, 0.94)	0.042^*∗*^

Baseline CD4 counts (cells/mm^3^)	<200	0.22 (0.03, 1.73)	0.4 (0.03, 6.04)	0.508
	200–349	0.12 (0.01, 1.004	0.15 (0.01, 2.33)	0.175
	350–499	0.15 (0.02, 1.35)	0.12 (0.007, 2.18)	0.152
	>500	1.00	1.00	

Current CD4 counts(cells/mm^3^)	<200	0.34 (0.16, 0.73)	0.03 (0.01, 0.160)	<0.001^*∗*^
	200–349	0.53 (0.29, 0.99)	0.57 (0.20, 1.63)	0.294
	350–499	1.15 (0.61, 2.18)	2.23 (0.84, 5.91)	0.106
	>500	1.00	1.00	

Current viral load (copies/ml)	≤1000 counts	1.00	1.00	
	≥1001 counts	0.50 (0.24, 1.04)	1.01 (0.26, 3.96)	0.987

Body mass index (BMI)		1.23 (1.15, 1.32)	3.50 (1.36, 3.89)	<0.001^*∗*^
Smoked cigarette	Yes	1.46 (0.48, 4.43)	1.32 (0.25, 7.12)	0.745
	No	1.00	1.00	

Consumed alcohol	Yes	0.86 (0.55, 1.34)	1.45 (0.66, 3.17)	0.351
	No	1.00	1.00	

Physical activity	Yes	1.00	1.00	0.86
	No	0.81 (0.50, 1.29)	0.93 (0.399, 2.16)	

Dietary diversity score	Diversified	1.05 (0.68, 1.61)	1.38 (0.65, 2.90)	0.40
	Undiversified	1.00	1.00	

Depression status	Normal	1.00	1.00	
	Abnormal	0.76 (0.43, 1.35)	0.58 (0.19, 1.74)	0.333

Total cholesterol (TC)	Normal	1.00	1.00	
	Abnormal	0.54 (0.35, 0.84)	0.39 (0.13, 1.22)	

Low-density lipoprotein (LDL)	Normal	1.00	1.00	
	Abnormal	1.83 (1.15, 2.89)	1.43 (0.46, 4.45)	0.539

High-density lipoprotein (HDL)	Normal	1.00	1.00	
	Abnormal	1.62 (0.98, 2.69)	1.15 (0.46, 2.84)	0.763

Total triglyceride (TG)	Normal	1.00	1.00	
	Abnormal	1.41 (0.83, 2.40)	1.59 (0.66, 3.79)	0.298

Blood pressure (BP)	Normal	1.00	1.00	
	Abnormal	1.55 (0.95, 2.54)	2.53 (1.13, 5.67)	0.024^*∗*^

Fasting blood glucose (FBS)		1.002 (0.99, 1.01)	1.004 (0.99, 1.02)	0.514

^*∗*^*p* value <0.05 (statistically significant association); 1.00: reference category. Hosmer and Lemeshow test (*p* value: 0.57).

## Data Availability

The dataset used for this study cannot be shared and, in the future, interested parties may request approval to access the data by writing to Jimma University Institutional Review Board.

## References

[1] Sabin C. A. (2013). Do people with HIV infection have a normal life expectancy in the era of combination antiretroviral therapy?. *BMC Medicine*.

[2] FHAPCO (2020). *HIV Prevention in Ethiopia National Road Map 2018–2020*.

[3] Tate T., Willig A. L., Willig J. H. (2013). HIV infection and obesity: where did all the wasting go. *Antiviral Therapy*.

[4] Nduka C. U., Uthman O. A., Kimani P. K., Stranges S. (2016). Body fat changes in people living with HIV on antiretroviral therapy. *AIDS Reviews*.

[5] Shah K., Alio A. P., Hall W. J., Luque A. E. (2012). The physiological effects of obesity in HIV-infected patients AIDS & clinical research. *Journal of AIDS & Clinical Research*.

[6] Thompson-Paul A. M., Wei S. C., Mattson C. L., Robertson M., Hernandez-Romieu A. C., Bell T. K. (2015). Obesity among HIV-infected adults receiving medical care in the United States: data from the cross-sectional medical monitoring project and national health and nutrition examination survey. *Medicine*.

[7] Dolan S. E., Hadigan C., Killilea K. M. (2005). Increased cardiovascular disease risk indices in in HIV-infected women. *Journal of Acquired Immune Deficiency Syndromes*.

[8] Justman J. E., Hoover D. R., Shi Q. (2015). Longitudinal anthropometric patterns among HIV-infected and—uninfected women jessica. *Journal of Acquired Immune Deficiency Syndromes*.

[9] Leite L. H. M., Sampaio A. B. D. M. M. (2010). Progression to overweight, obesity and associated factors after antiretroviral therapy initiation among brazilian persons with HIV/AIDS. *Nutricion Hospitalaria*.

[10] Noorhasan M., Drozd D. R., Grunfeld C. (2017). Associations between at-risk alcohol use, substance use, and smoking with lipohypertrophy and lipoatrophy among patients living with HIV. *AIDS Research and Human Retroviruses*.

[11] Njelekela M., Mpembeni R., Muhihi A., Ulenga N., Aris E., Kakoko D. (2017). Lipodystrophy among HIV-infected patients attending care and treatment clinics in dar es salaam. *AIDS Research and Treatment*.

[12] Hejazi N. (2010). Factors associated with abdominal obesity among HIV-infected adults on antiretroviral therapy in Malaysia. *Global Journal of Health Science*.

[13] Florindo A. A., Latorre O. (2006). Central obesity and dietary intake in HIV/AIDS patients obesidade abdominal e consumo alimentar em portadores de HIV/. *Revista de Saúde Pública*.

[14] Castro A. d. C. O., Silveira E. A., Falco M. d. O., Nery M. W., Turchi M. D. (2016). Overweight and abdominal obesity in adults living with HIV/AIDS. *Revista da Associação Médica Brasileira*.

[15] Khetsiwe S., Masuku S., Gwegweni J. T., Sartorius B. (2019). HIV and antiretroviral therapy—induced metabolic syndrome in people living with HIV and its implications for care: a critical review. *Journal of Diabetology*.

[16] Kumar S., Samaras K. (2018). The impact of weight gain during HIV treatment on risk of pre-diabetes, diabetes mellitus, cardiovascular disease, and mortality. *Frontiers in Endocrinology*.

[17] Neves J. S., Guerreiro V., Carvalho D., Serrão R. (2018). Metabolically healthy or metabolically unhealthy obese HIV-infected patients: mostly a matter of age?. *Frontiers in Endocrinology*.

[18] Lake J. E. (2017). The fat of the matter: obesity and visceral adiposity in treated HIV infection. *Current HIV*.

[19] Papagianni M., Tziomalos K. (2016). Obesity in patients with HIV infection: epidemiology, consequences and treatment options. *Expert Review of Endocrinology and Metabolism*.

[20] Finkelstein J. L., Gala P., Rochford R., Glesby M. J., Mehta S. (2015). HIV/AIDS and lipodystrophy: implications for clinical management in resource-limited settings. *Journal of the International AIDS Society*.

[21] Dimala C. A., Ngu R. C., Kadia B. M., Tianyi F. L., Choukem S. P. (2018). Markers of adiposity in HIV/AIDS patients: agreement between waist circumference, waist-to-hip ratio, waist-to-height ratio and body mass index. *PLoS One*.

[22] Onesi S. O., Ignatius U. E. (2014). Metabolic syndrome: performance of five different diagnostic criterias. *Indian Journal of Endocrinology and Metabolism*.

[23] Alberti K. G. M. M., Zimmet P., Shaw J. (2006). Metabolic syndrome-a new world-wide definition. a consensus statement from the international diabetes federation. *Diabetic Medicine*.

[24] WHO (2005). *The WHO STEPwise Approach to Chronic Disease Risk Factor Surveillance*.

[25] Stomdfacpfac E., Kaufman J. S., Bella A. F. (2018). Central obesity in Africans: anthropometric assessment of abdominal adiposity and its predictors in urban Nigerians. *Journal of the National Medical Association*.

[26] Saito A., Karama M., Kamiya Y. (2020). HIV infection, and overweight and hypertension: a cross-sectional study of HIV-infected adults in Western Kenya. *Tropical Medicine and Health*.

[27] Expert W. (2008). *Waist Circumference and Waist-Hip Ratio Report of a WHO Expert Consultation*.

[28] Ngu R. C., Choukem S. P., Dimala C. A., Ngu J. N. (2018). Prevalence and determinants of selected cardio—metabolic risk factors among people living with HIV/AIDS and receiving care in the South West regional hospitals of cameroon: a cross—sectional study. *BMC Research Notes*.

[29] Nurdiantami Y., Watanabe K., Tanaka E., Pradono J., Anme T. (2017). Association of general and central obesity with hypertension. *Clinical Nutrition*.

[30] Choudhury K. N., Mainuddin A. K., Wahiduzzaman M., Islam S. M. S. (2014). Serum lipid profile and its association with hypertension in Bangladesh. *Vascular Health and Risk Management*.

[31] Abhijit Swami D. S. K. (2017). A study of metabolic syndrome in HIV patients treated with NNRTI based antiretroviral therapy. *Scholars Journal of Applied Medical Sciences*.

[32] Paula A. A., Falcão M. C. N., Pacheco A. G. (2013). Metabolic syndrome in HIV-infected individuals: underlying mechanisms and epidemiological aspects. *AIDS Research and Therapy*.

